# Atorvastatin Represses the Angiotensin 2-Induced Oxidative Stress and Inflammatory Response in Dendritic Cells via the PI3K/Akt/Nrf 2 Pathway

**DOI:** 10.1155/2014/148798

**Published:** 2014-07-03

**Authors:** Yuanji Ma, Zhaoyang Chen, Yunzeng Zou, Junbo Ge

**Affiliations:** ^1^Shanghai Institute of Cardiovascular Diseases, Zhongshan Hospital, Fudan University, 180 Fenglin Road, Shanghai 200032, China; ^2^Institute of Biomedical Science, Fudan University, 138 Yixueyuan Road, Shanghai 200032, China

## Abstract

Dendritic cells (DCs), which are highly proficient antigen-presenting cells, play a complex role in both the initiation and progression of atherosclerosis. We tested the hypothesis that the anti-inflammatory and antioxidant effects of atorvastatin may be partly mediated by the phosphatidylinositol 3-kinase/protein kinase B/transcription factor nuclear factor-erythroid 2-related factor 2 (PI3K/Akt/Nrf 2) pathway via the attenuation of DC maturation, thus reducing the inflammatory and oxidative stress responses. This study showed that angiotensin 2 (Ang 2) induced the maturation of DCs, stimulated CD83, CD40, CD80, and CD86 expression, and increased the secretion of IL-12p70, IL-6, and TNF-*α*. These effects were suppressed by atorvastatin. Atorvastatin also lowered the levels of reactive oxygen species (ROS) and malondialdehyde (MDA), counteracting their initial increases in response to Ang 2 stimulation. Atorvastatin activated Nrf 2 via the PI3K/Akt pathway and thereby promoted Nrf 2 translocation from the cytoplasm to the nucleus in bone marrow-derived dendritic cells (BMDCs), a process that was reversed by the PI3K inhibitor LY294002. Therefore, the regulation of Nrf 2 expression by the PI3K/Akt pathway plays an important role in the regulation of the statin-mediated antioxidant and anti-inflammatory responses in DCs.

## 1. Introduction

Atherosclerosis (AS) is a chronic, multifactorial disease that develops in response to inflammation and oxidative stress triggered by immune responses to autoantigens or by cross-reactions to foreign antigens, triggering the formation of lesions in arterial blood vessels [1]. Dendritic cells (DCs) are antigen-presenting cells that are highly involved in the process of AS, an involvement characterized by the activation of T cells and the stimulation of vascular inflammatory responses [2, 3]. The maturation of DCs is stimulated by angiotensin 2 (Ang 2), oxidized low-density lipoprotein (oxLDL), advanced glycosylation end products [3], or other antigens associated with atherosclerotic lesions [4–6], the development of which is characterized by an increasing amount of cytokine secretions and the upregulation of CD83, CD80, CD86, and CD40 [7]. Ang 2, one of the most important factors in the development of AS, mediates many cellular activities by stimulating the formation of reactive oxygen species (ROS), and it also initiates inflammatory responses by activating DCs [4, 8]. Several studies have shown that statins exert many effects beyond lowering cholesterol to ameliorate endothelial function, such as by increasing atherosclerotic plaque stability and activating anti-inflammatory and antioxidant mediators [9–11].

Transcription factor nuclear factor-erythroid 2-related factor 2 (Nrf 2), a regulator of cellular oxidative stress [12] and inflammatory response activation [13], is a widely expressed transcription factor during AS. When exposed to ROS or other inducers, Nrf 2 is released from Kelch-like ECH-associated protein 1 (Keap 1) and translocates into the nucleus, where it induces the expression of antioxidant genes by binding to antioxidant response elements (AREs) [14], driving the transcription of many antioxidative genes and protecting cells from oxidative stress. Previous evidence showed that the phosphatidylinositol 3-kinase/protein kinase B (PI3K/Akt) pathway plays a role in regulating Nrf 2 activation and its subsequent nuclear translocation [15]. Moreover, the phosphorylation of Akt has been associated with the activation of Nrf 2 [16].

Our previous studies have suggested that the immune maturation of DCs may play a crucial role in atherosclerotic lesions and that DC suppression may be involved in antiatherogenic mechanisms [6, 17, 18]. Furthermore, several recent studies have shown that statins activate the PI3K/Akt pathway, resulting in the upregulation of Nrf 2 [19, 20]. However, the precise biochemical and molecular mechanisms by which statins inhibit oxidative stress and inflammation are not completely understood. Therefore, the purpose of this study was to investigate the mechanisms regulating oxidative stress and inflammatory responses in DCs.

## 2. Materials and Methods

### 2.1. Cell Culture and Treatments

Bone marrow-derived dendritic cells (BMDCs) obtained from approximately 6-week-old C57BL/6 mice were cultured in RPMI 1640 media supplemented with 10 ng/mL granulocyte-macrophage colony-stimulating factor (GM-CSF) and 1 ng/mL IL-4 at 37°C in 5% humidified CO_2_ for 4 h. Medium containing nonadherent cells was replaced with fresh medium every 2 days. On culture day 7, the cells were treated with either Ang 2 (100 nM) (Sigma-Aldrich, St. Louis, MO, USA) alone or in combination with various concentrations (2.5, 5, or 10 *μ*M) of atorvastatin (Sigma-Aldrich, St. Louis, MO, USA) for 24 h. Phosphate-buffered saline (PBS) was used as a control. In the inhibitor experiment, cells were exposed to Ang 2 (100 nM) for 24 h after pretreatment with an inhibitor (LY294002, 100 nM) for 1 h, whereas in the Nrf 2 activator experiment, cells were exposed to Ang 2 (100 nM) for 24 h after pretreatment with either 10 *μ*M atorvastatin or 10 *μ*M sulforaphane (SUL, Sigma-Aldrich, St. Louis, MO, USA) for 1 h. The antibodies for Akt, phospho-Akt (Ser473), and Nrf 2 (Ser40), as well as the inhibitor LY294002, were purchased from Cell Signaling Technology (Beverly, MA, USA).

### 2.2. Flow Cytometric Measurement

BMDCs were washed and resuspended in ice-cold PBS containing 5% fetal bovine serum to prevent nonspecific binding and incubated with anti-CD83, anti-CD80, anti-CD86, and anti-CD40 (BD Pharmingen, San Diego, CA, USA) for 30 minutes at 4°C. After extensive washing, the stained cells were analyzed using a FACScan flow cytometer (BD Biosciences, San Jose, CA, USA) and Cell Quest software.

Intracellular levels of ROS were measured with DCFH-DA molecular probes (Molecular Probes-Invitrogen, Carlsbad, CA, USA). Cells were incubated with 10 *μ*M DCFH-DA for 30 min at 37°C and then washed and resuspended in PBS at 1 × 10^6^ cells/mL. The DCs were analyzed using flow cytometry. The fluorescence was determined at 503/529 nm and expressed as a percentage of the control.

### 2.3. Western Immunoblotting

Protein samples were fractionated with 12% SDS-PAGE (Invitrogen, Carlsbad, CA, USA) and transferred to polyvinylidene fluoride membranes (Millipore, Bedford, MA, USA). The membranes with blotted protein were blocked, followed by probing with Akt, phospho-Akt (Ser473), and Nrf 2 antibodies at 4°C overnight. The membranes were washed and incubated at room temperature for 2 h with diluted (1 : 5000) secondary HRP-conjugated antibodies. Immunoreactive proteins were identified using SuperSignal West Pico Chemiluminescent Substrate (Thermo, Franklin, MA, USA). Densitometric analysis of the western blotting was performed using Image J software. *β*-Actin was used as the loading control.

### 2.4. Enzyme-Linked Immunosorbent Assay

The supernatant of the cultured BMDCs was harvested and stored at −70°C. The cytokine concentrations of TNF-*α*, IL-12P40, IL-6, and IFN-*γ* were analyzed using enzyme-linked immunosorbent assay (ELISA) kits (R&D Systems, Minneapolis, MN, USA) according to the manufacturer's instructions. The superoxide dismutase (SOD) activity and MDA contents were measured at 450 and 532 nm by SOD and MDA ELISA kits (R&D Systems, Minneapolis, MN, USA), respectively.

### 2.5. T Cell Proliferation Assays

The effects of atorvastatin on T cell proliferation mediated by Ang 2-treated BMDCs were analyzed in a mixed lymphocyte reaction. Adult T cells from the spleens of C57BL/6 mice were purified using a Dynal Mouse T Cell Negative Isolation Kit (Invitrogen, Paisley, UK). BMDCs were added to 1 × 10^5^ T cells at a ratio of 1 : 10 (DCs : T cells) and incubated for 5 days in 96-well tissue culture plates. The cells were incubated with 20 *μ*L of diluted bromodeoxyuridine (BrdU) (Chemicon, Temecula, CA, USA) for 18 h and were then analyzed on a spectrophotometer microplate reader at 490 nm, according to the manufacturer's instructions.

### 2.6. Statistical Analyses

The data are presented as the means ± SD, with *P* < 0.05 considered to be statistically significant. A one-way ANOVA, followed by the Student-Newman-Keuls test, was employed for the statistical analysis of our results. All statistical analyses were performed with SPSS 11.5 statistical software.

## 3. Results

### 3.1. Atorvastatin Attenuates Ang 2-Induced Oxidative Stress in BMDCs

**Figure 1:**
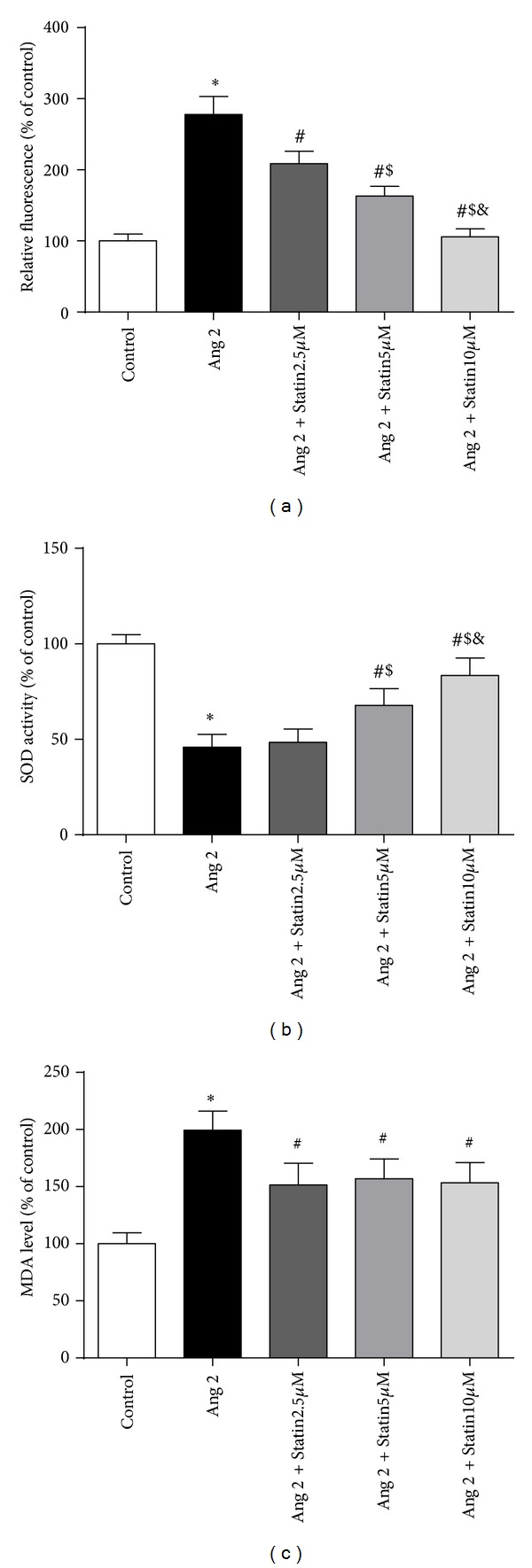
Effects of atorvastatin on ROS formation (a), SOD activity (b), and MDA levels (c) in BMDCs in the presence of Ang 2. BMDCs were incubated with Ang 2 (100 nM) alone or in combination with various concentrations (2.5, 5, or 10 *μ*M) of atorvastatin for 24 h. (a) The intracellular ROS levels were measured via flow cytometry using DCFH-DA. (b) The SOD activity and (c) MDA levels were measured at 450 and 532 nm, respectively, using ELISA kits. The data are shown as the mean ± (SD) (*n* = 3); *P < 0.05 versus control; ^#^ *P* < 0.05 versus Ang 2 group; ^$^ *P* < 0.05 versus Ang 2 + 2.5 *μ*M atorvastatin group; ^&^ *P* < 0.05 versus Ang 2 + 5 *μ*M atorvastatin group.

To investigate how atorvastatin affects Ang 2-induced oxidative stress in BMDCs, we used flow cytometry to measure the intracellular level of ROS in response to Ang 2 stimulation, and we also measured the levels of MDA and SOD with ELISA kits. As depicted in Figure 1, 100 nM Ang 2 exhibited a stimulating effect on ROS (a) and MDA production (c) but inhibited SOD activity. Cotreatment with atorvastatin with concentrations ranging from 2.5 to 10 *μ*M reduced intracellular ROS production in a dose-dependent manner, and similar results were observed with MDA. However, atorvastatin suppressed the Ang 2-induced inhibition of SOD activity (b). Therefore, these results suggest that atorvastatin significantly suppresses Ang 2-induced oxidative stress in BMDCs.

### 3.2. Effect of Atorvastatin on Ang 2-Induced BMDC Maturation and Inflammatory Cytokine Secretion

**Figure 2:**
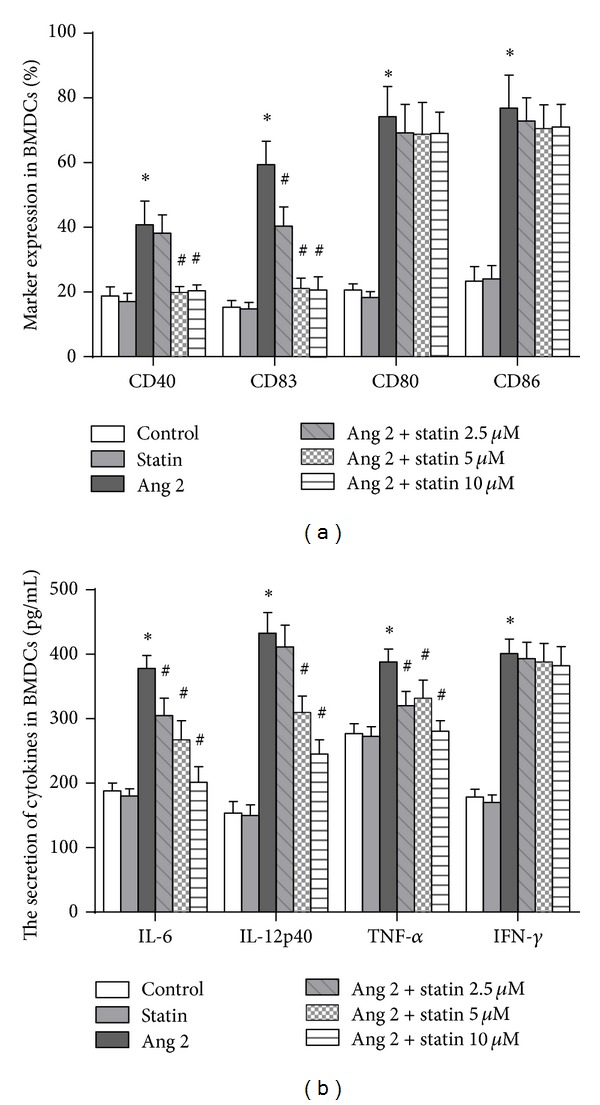
Inhibitory effects of atorvastatin on Ang 2-induced BMDC maturation and inflammatory responses. After pretreatment with either PBS or Ang 2 (100 nM), BMDCs were exposed to various concentrations of atorvastatin (2.5, 5, or 10 *μ*M) for 24 h. (a) Expression of the cell-surface markers CD40, CD83, CD80, and CD86, as determined by flow cytometry (*n* = 3). (b) Expression of cytokines in BMDCs analyzed by ELISA (*n* = 3). The data are shown as the mean ± (SD) (*n* = 3); *P < 0.05 versus control; ^#^ *P* < 0.05 versus Ang 2 group.

There are many costimulatory proteins on the surface of BMDCs, including CD40, CD83, CD80, and CD86. Therefore, these proteins were examined in Ang 2-treated (100 nM) BMDCs that were exposed to various concentrations of atorvastatin (2.5, 5, or 10 *μ*M). The flow cytometry results showed that the expression of the cell-surface markers CD40, CD83, CD80, and CD86 was upregulated by Ang 2. We subsequently observed that atorvastatin significantly downregulates CD83 and CD40 expression but exerts moderate inhibitory effects on CD86 and CD80. These results indicate that atorvastatin may suppress Ang 2-induced DC maturation (Figure 2(a)).

An analysis of cytokine levels, that is, TNF-*α*, IL-12P40, IL-6, and IFN-*γ*, indicated an Ang 2-induced inflammatory response in BMDCs. We found that the secretion of TNF-*α*, IL-6, and IL-12p70 was increased significantly in the presence of Ang 2. In addition, the secretion of these cytokines was suppressed by atorvastatin in a dose-dependent manner. However, IFN-*γ* was clearly not affected by either Ang 2 or atorvastatin (Figure 2(b)).

### 3.3. Atorvastatin Activates Nrf 2 through the PI3K/Akt Pathway in BMDCs

**Figure 3:**
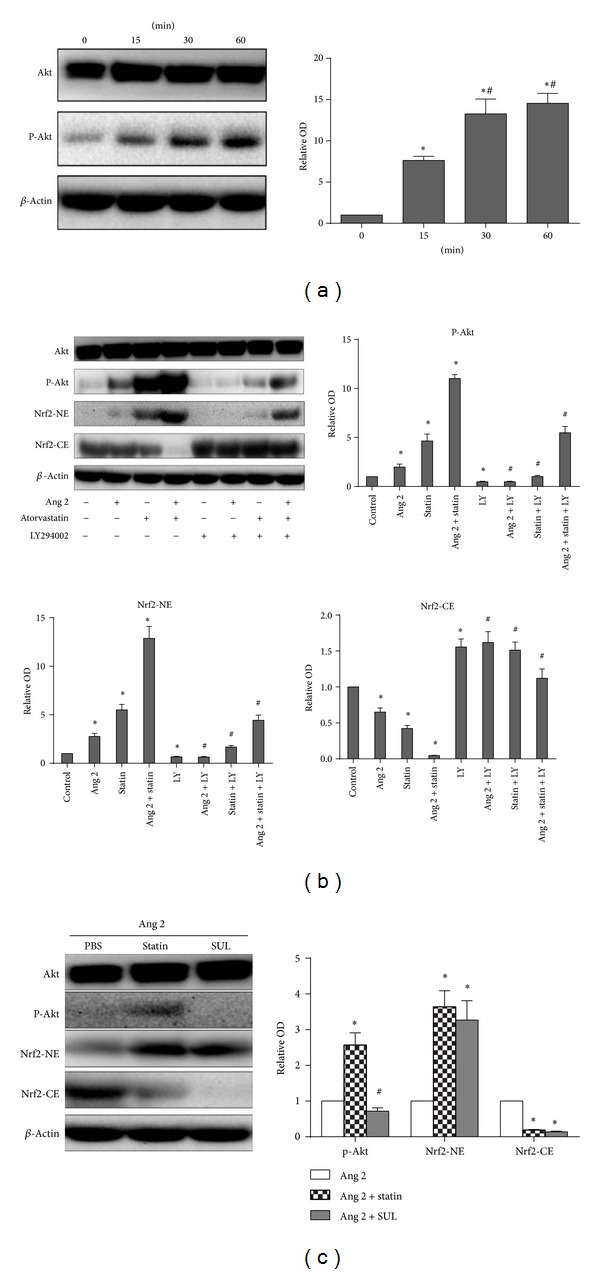
PI3K/Akt/Nrf 2 pathway activation mediates the protective effect of atorvastatin in BMDCs. (a) Atorvastatin promotes Akt phosphorylation in a time-dependent manner. BMDCs were treated with 10 *μ*M atorvastatin for the indicated times. *β*-Actin was employed as a loading control. The graph represents the means ± (SD) (*n* = 3), *P < 0.05 versus time 0 min; ^#^ *P* < 0.05 versus time 15 min. (b) The nuclear translocation of Nrf 2 was mediated by PI3K/Akt signals in atorvastatin-treated cells. BMDCs were treated with 100 nM Ang 2 in the presence or absence of 10 *μ*M atorvastatin. Cellular cytoplasmic extracts (CE) and nuclear extracts (NE) were separated on a 12% SDS-PAGE gel to probe Nrf 2 and actin levels by western blotting. Total extracts of the cells were prepared, and the p-Akt (Ser473) expression was assayed by western immunoblotting. In the presence of 100 nM LY294002, the effects of atorvastatin on both Akt phosphorylation and Nrf 2 translocation were inhibited. (c) Nrf 2 activation was involved in the atorvastatin-mediated inhibition of Ang 2-induced oxidative stress. Atorvastatin induced the translocation of Nrf 2 into the nucleus and the phosphorylation of Akt. However, with 10 *μ*M SUL, we observed only Nrf 2 activation and not Akt phosphorylation. *β*-Actin was employed as a loading control. The data represent the means ± SD (*n* = 3) from three independent repeats; *P < 0.05 versus control; ^#^ *P* < 0.05 versus Ang 2 + atorvastatin group.

Recent studies have demonstrated that statins activate the PI3K/Akt pathway [19, 20]. To confirm these findings, we monitored the phosphorylation of Akt at position Ser473 by western blotting in atorvastatin-treated BMDCs. Surprisingly, as shown in Figure 3(a), we found that atorvastatin promoted Akt phosphorylation in a time-dependent manner.

Nrf 2 plays a central role in the cellular antioxidative system. The nuclear accumulation of Nrf 2 is considered to be a marker of Nrf 2 activation in response to stressors. Moreover, the PI3K/Akt pathway is involved in regulating Nrf 2 activation and the subsequent nuclear translocation of Nrf 2 [15]. Therefore, we further investigated the molecular mechanisms underlying the effects of atorvastatin on Nrf 2 activation. To address this question, BMDCs were treated with 100 nM Ang 2 in the presence or absence of 10 *μ*M atorvastatin, and the cell lysate was fractionated into cytoplasmic and nuclear fractions. The fractions were then analyzed by western blotting for Nrf 2. Western blotting (Figure 3(b)) revealed that the level of Nrf 2 in the cytoplasmic fraction was significantly reduced, compared with the control, by both atorvastatin alone and atorvastatin in combination with Ang 2. By contrast, the level of Nrf 2 in the nuclear fraction was increased under the same conditions. These results demonstrate that atorvastatin promoted the nuclear translocation of Nrf 2 in Ang 2-treated BMDCs, by increasing both the dissociation of Nrf 2 from Keap 1 and the translocation of Nrf 2 to the nucleus [14].

Next, we used LY294002 (100 nM), a PI3K inhibitor, to assess whether this pathway is involved in Nrf 2 activation by atorvastatin. Western blot analysis showed that, in Ang 2-treated BMDCs, atorvastatin promoted the phosphorylation of Akt and the translocation of Nrf 2. Treatment of the cells with the PI3K inhibitor LY294002 blocked these effects.

To further confirm the involvement of Nrf 2 activation in atorvastatin-mediated actions against Ang 2-induced oxidative stress, we used the Nrf 2 activator SUL to evaluate whether atorvastatin has an effect on the translocation of Nrf 2 into the nucleus in BMDCs. As shown in Figure 3(c), western blotting analysis confirmed that similar to SUL, which is a known Nrf 2 activator, atorvastatin induced the translocation of Nrf 2 into the nucleus. However, while atorvastatin promoted Akt phosphorylation, SUL did not. These results, which are shown in Figure 3, indicate that the PI3K/Akt/Nrf 2 pathway is involved in the atorvastatin-mediated inhibition of Ang 2-induced cellular responses.

### 3.4. PI3K/Akt Pathway Involvement in Ang 2-Induced Oxidative Stress and Inflammatory Responses Is Suppressed by Atorvastatin

**Figure 4:**
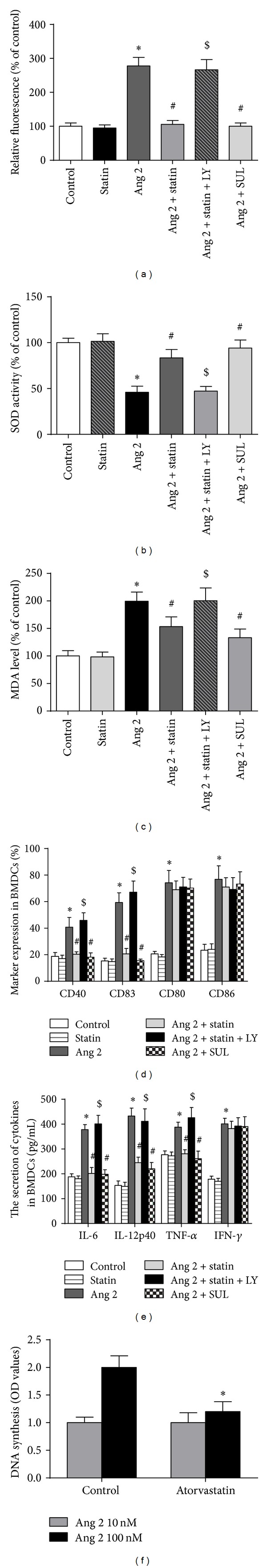
The PI3K/Akt/Nrf 2 pathway is involved in the atorvastatin-mediated inhibition of Ang 2-induced cellular responses. After pretreatment with PBS and either the Nrf 2 activator SUL (10 *μ*M) or the PI3K inhibitor LY294002 (100 nM) for 1 h, BMDCs were incubated for 24 h with Ang 2 (100 nM) alone, atorvastatin (10 *μ*M) alone, or Ang 2 (100 nM) and atorvastatin (10 *μ*M) combined. We observed markers of oxidative stress and inflammation in the BMDCs. (a) Intracellular ROS levels were measured with flow cytometry using DCFH-DA. (b) SOD activity and (c) MDA levels were measured by ELISA kits. (d) Expression of the cell-surface markers CD40, CD83, CD80, and CD86 as determined by flow cytometry (*n* = 3). (e) Expression of the cytokines in BMDCs analyzed by ELISA (*n* = 3). (f) Atorvastatin decreased the ability of BMDCs to activate T cells. BMDCs were cultured with 100 nM Ang 2 for 48 h in the absence or presence of 10 *μ*M atorvastatin. Cells were harvested and then incubated with 1 × 10^5^ T cells in a 96-well plate at a ratio of 1 : 10 (BMDCs : T cells) for 5 days. For the final 18 h of incubation, 20 *μ*L of diluted BrdU was added to the appropriate wells (*n* = 3). The data are shown as the means ± (SD) (*n* = 3); *P < 0.05 versus control; ^#^ *P* < 0.05 versus Ang 2 group; ^$^ *P* < 0.05 versus Ang 2 + atorvastatin group.

Because atorvastatin activates the PI3K/Akt/Nrf 2 pathway, we investigated whether atorvastatin influences the course of Ang 2-induced oxidative stress and inflammatory responses. We found that atorvastatin and SUL had inhibitory effects on ROS and MDA production but stimulated SOD activity. In addition, the PI3K inhibitor LY294002 significantly diminished the antioxidant effects of atorvastatin (Figures 4(a), 4(b), and 4(c)).

We next studied the atorvastatin-mediated inhibition of Ang 2-induced inflammation. We found that SUL had the same effect on DC maturation and inflammatory cytokines as atorvastatin. However, the pretreatment of cells with 100 nM LY294002 abrogated the influence of atorvastatin on DC maturation and inflammatory cytokine mediators, such as CD83, CD40, TNF-*α*, IL-6, and IL-12p70 (Figures 4(d) and 4(e)). These results indicate the involvement of the PI3K/Akt/Nrf 2 pathway in BMDC maturation and inflammation.

### 3.5. Atorvastatin Attenuated the Ability of Ang 2-Treated BMDCs to Induce T Cell Proliferation

To further demonstrate the physiological relevance of our findings, we evaluated the effect of atorvastatin on antigen presentation. As shown in Figure 4(f), T cell proliferation was detected by BrdU, and BMDCs exposed to Ang 2 effectively induced allogeneic T cell proliferation compared with controls. However, 10 *μ*M atorvastatin almost completely blocked Ang 2-induced T cell activation.

## 4. Discussion

The major finding of our study is that atorvastatin represses the oxidative stress induced by Ang 2 in BMDCs via the PI3K/Akt/Nrf 2 pathway. Atorvastatin also inhibited Ang 2-induced inflammatory responses via the downregulation of BMDC surface markers and inflammatory cytokines. Additionally, blocking the PI3K/Akt/Nrf 2 pathway may abrogate the effects of atorvastatin on BMDCs during inflammation and oxidative stress.

Previous studies by our group indicated that mature DCs may play key roles in the initiation and regulation of immune responses involved in atherogenesis, as evidenced by the antiatherogenic effects of DC suppression [6, 17, 18]. Statins, the cornerstone of antiatherogenic therapy, have an effect on DC maturation [21], but the mechanisms underlying this effect remain unclear. In this study, we focused on the impact of Ang 2 on DC activation. In doing so, we investigated the influence of atorvastatin on DC maturation and cytokine production induced by Ang 2. CD83, CD40, and CD80/CD86 are characteristic surface markers in mature DCs. CD83 is preferentially expressed on mature DCs [22], whereas CD40 is a member of the TNF receptor family, which is transiently expressed on T cells under inflammatory conditions [23]. We found that atorvastatin downregulates the expression of both CD83 and CD40, the expression of which is stimulated by Ang 2. This indicates that atorvastatin attenuates atherosclerotic lesions by downregulating DC surface molecules. Additionally, atorvastatin appears to inhibit DC maturation and antigen uptake and presentation, which are induced by LPS, Ang 2, and proinflammatory factors, and facilitate an antigen-specific T cell immune response [24, 25]. In addition, we found that statins suppress Ang 2-induced allogeneic T cell proliferation. Finally, the inflammatory activation of DCs is also reflected by the differential expression of the cytokines induced by Ang 2. We discovered that Ang 2 increased the expression of the proinflammatory cytokines IL-12p70, IL-6, and TNF-*α*. By contrast, atorvastatin inhibited this Ang 2-induced increase. Surprisingly, all of these effects on both DC maturation and inflammation were reversed following the addition of the PI3K inhibitor, LY294002. Therefore, the PI3K/Akt pathway is involved in the atorvastatin-mediated suppression of DC maturation and inflammation.

SUL activity is explained primarily by its activation of the Nrf 2-ARE pathway [26]. In this study, the administration of SUL resulted in a significant increase in the translocation of Nrf 2 to the nucleus without activating Akt. After SUL administration, the release of the inflammatory cytokines IL-12p70, IL-6, and TNF-*α* and the expression of the surface markers CD83 and CD40 were attenuated. The effect of SUL was reflected further by the augmentation of SOD activity with an accompanying decrease in both the MDA and ROS levels. SUL enhanced the activity of the Nrf 2-ARE pathway, suppressing the inflammatory responses and oxidative stress induced by Ang 2.

Oxidative stress resulting from uncontrolled ROS production has been implicated in the pathogenesis of atherosclerosis. Several studies have explained the potential beneficial pleiotropic and antioxidant effects of statins [9–11]. The PI3K/Akt/Nrf 2 pathway has been shown to be involved in the antioxidant effects of statins [19, 20]. However, the effects and mechanisms by which statins interact with DCs are less well studied, particularly where antioxidants are concerned. In this study, we showed that atorvastatin lowers ROS and MDA levels via regulation of the PI3K/Akt/Nrf 2 pathway.

To mitigate the cumulative burden of oxidative stress, cells generally utilize antioxidant defense systems and scavenge ROS and MDA. SOD is an important antioxidant enzyme. Our study indicated that DCs treated with Ang 2 showed a marked increase in oxidative stress, as evidenced by excessive ROS and MDA production. However, cotreatment with atorvastatin significantly attenuated oxidative damage induced by Ang 2, as reflected by the augmentation of SOD activity and the accompanying decrease in MDA and ROS levels. There are a few mechanisms by which Ang 2 could downregulate SOD activity. The inhibition of small G proteins, such as the NADPH oxidase component, Rac1 [27, 28], and the reduced expression of NADPH oxidase subunits [28], may mediate the antioxidant effects of atorvastatin. Ang 2 triggers NADH/NADPH-oxidase and local vascular O_2^.^_^−^ production [29–31]. It was recently demonstrated that Ang 2 increases Mn-SOD, diminishes CuZn-SOD, and does not alter EC-SOD expression [32]. In our study, atorvastatin treatment reversed the alterations observed during oxidative stress, a reversal most likely due to an increase in antioxidant defenses and diminished NADPH oxidase expression. In addition, we observed increased SOD activity with atorvastatin treatment.

Additionally, Nrf 2 is a regulator of cellular oxidative stress and inflammatory activity. It is sequestered in the cytoplasm as an inactive complex with its cytosolic repressor, Keap 1. The dissociation of Nrf 2 from Keap 1 is a prerequisite for the nuclear translocation and subsequent DNA binding of Nrf 2 [14]. The dissociation of the Nrf 2-Keap 1 complex, which is mediated via one or more upstream kinases, including MAPKs, PI3K/Akt, and PKC, was recently reviewed [15, 16, 33]. We confirmed that atorvastatin activates the PI3K/Akt pathway, further showing that this pathway is a prerequisite for the activation of Nrf 2. Atorvastatin promotes both Akt phosphorylation and Nrf 2 translocation in Ang 2-treated DCs. We also observed Nrf 2 translocation, but not Akt phosphorylation, after replacing atorvastatin with the Nrf 2 activator SUL. Moreover, the PI3K inhibitor LY294002 significantly repressed the stimulatory effects of atorvastatin on Nrf 2 activation. Therefore, these results show that the PI3K/Akt pathway partially regulates Nrf 2 translocation, which is consistent with a prior finding in H9c2 cardiomyocytes [34], thereby regulating oxidative stress and inflammation. It seems probable that atorvastatin activates the PI3K/Akt pathway via Akt phosphorylation at position Ser473, mediating Nrf 2 activation.

Blocking the PI3K/Akt/Nrf 2 pathway may repress not only the inflammatory reactions but also the oxidative stress induced by Ang 2. Oxidative signals play crucial roles in the pathogenesis of chronic inflammatory diseases by mediating the expression of inflammatory genes, such as MCP-1 and VCAM-1. Previous studies have demonstrated that the expression of Nrf 2 suppresses TNF-*α*-induced MCP-1 and VCAM-1 expression, monocyte adhesion to endothelial cells, and the activation of p38 MAPK [35]. Nrf 2 also regulates a set of antioxidant proteins, such as heme oxygenase-1 (HO-1), that includes important factors for modulating inflammatory responses [36]. The overexpression of HO-1 has been shown to suppress oxidized LDL-induced monocyte transmigration, inhibiting atherosclerotic lesion formation in LDL-receptor knockout mice [37, 38]. The coordinated regulation of these genes has synergistic effects on the maintenance of intracellular antioxidant capacity and the inhibition of inflammatory responses. However, further research of the mechanisms mediating inflammation and oxidative stress is still needed.

In summary, our results demonstrate that atorvastatin inhibits the oxidative stress and inflammatory responses induced by Ang 2 via the PI3K/Akt/Nrf 2 pathway. Furthermore, the activation of Nrf 2 by atorvastatin may be a means of both antioxidant protection and the suppression of redox-sensitive inflammatory genes, suggesting that targeting Nrf 2 activation may represent a novel therapeutic approach in the treatment of atherosclerosis.

## Abbreviations

DC: Dendritic cell
AS: Atherosclerosis
Ang 2: Angiotensin 2
ROS: Reactive oxygen species
MDA: Malondialdehyde
oxLDL: Oxidized low-density lipoprotein
Nrf 2: Transcription factor nuclear factor-erythroid 2-related factor 2
Keap 1: Kelch-like ECH-associated protein 1
ARE: Antioxidant response element
PI3K: Phosphatidylinositol 3-kinase
Akt: Protein kinase B
BMDCs: Bone marrow-derived dendritic cells
GM-CSF: Granulocyte-macrophage colony-stimulating factor
SUL: Sulforaphane
PBS: Phosphate-buffered saline
IL: Interleukin
ELISA: Enzyme-linked immunosorbent assay
BrdU: Bromodeoxyuridine
SOD: Superoxide dismutase
TNF-*α*: Tumor necrosis factor-*α*
IFN-*γ*: Interferon *γ*
CE: Cytoplasmic extracts
NE: Nuclear extracts
HO: Heme oxygenase
VCAM: Vascular cell adhesion molecule-1
MCP: Monocyte chemotactic protein
MAPK: Mitogen-activated protein kinase.
